# Phacoemulsification Combined With Supra-Capsular and Scleral-Fixated Intraocular Lens Implantation in Microspherophakia: A Retrospective Comparative Study

**DOI:** 10.3389/fmed.2022.869539

**Published:** 2022-04-14

**Authors:** Ze-Xu Chen, Zhen-Nan Zhao, Yang Sun, Wan-Nan Jia, Jia-Lei Zheng, Jia-Hui Chen, Tian-Hui Chen, Li-Na Lan, Yong-Xiang Jiang

**Affiliations:** ^1^Eye Institute and Department of Ophthalmology, Eye and ENT Hospital, Fudan University, Shanghai, China; ^2^NHC Key Laboratory of Myopia, Fudan University, Shanghai, China; ^3^Key Laboratory of Myopia, Chinese Academy of Medical Sciences, Shanghai, China; ^4^Shanghai Key Laboratory of Visual Impairment and Restoration, Shanghai, China

**Keywords:** microspherophakia, capsular bag, phacoemulsification, modified capsular tension ring, Nd:YAG laser capsulotomy

## Abstract

**Background:**

Microspherophakia (MSP) is a rare ocular condition, the lens surgery of which is complicated by both insufficient zonules and undersized capsule.

**Methods:**

This study included MSP eyes managed with phacoemulsification combined with supra-capsular and scleral-fixated intraocular lens implantation (SCSF-IOL) and made the comparison with those treated by transscleral-fixated modified capsular tension ring and in-the-bag intraocular lens implantation (MCTR-IOL).

**Results:**

A total of 20 MSP patients underwent SCSF-IOL, and 17 patients received MCTR-IOL. The postoperative best corrected visual acuity was significantly improved in both groups (*P* < 0.001), but no difference was found between the groups (*P* = 0.326). The IOL tilt was also comparable (*P* = 0.216). Prophylactic Nd:YAG laser posterior capsulotomy was performed 1 week to 1 month after the SCSF-IOL procedure. In the SCSF-IOL group, two eyes (10.00%) needed repeated laser treatment and one eye (5.00%) had a decentered capsule opening. Posterior capsule opacification was the most common complication (6, 35.29%) in the MCTR group. No IOL dislocation, secondary glaucoma, or retinal detachment was observed during follow-up.

**Conclusions:**

SCSF-IOL is a viable option for managing MSP and is comparable with the MCTR-IOL. Nd:YAG laser posterior capsulotomy was necessary to prevent residual capsule complications after the SCSF-IOL procedure.

## Introduction

Lens zonules are not only involved in accommodation by transferring the tensive force exerted by the ciliary body, but they also modulate the proliferation of lens epithelial cells (1). Microspherophakia (MSP) is a rare congenital abnormality in which lens growth is arrested by the lack of tension from rudimentary zonules. Without sufficient stretching, the lens fails to develop a biconvex shape and remains spherical at the fifth to the 6 month of embryonic life (2), in that the entire lenticular equator is visible under complete pupil dilation. As their lens is smaller and more spherically shaped, patients with MSP are often complicated by lens subluxation (44%) and high lenticular myopia (84.6%) and have a high propensity for secondary glaucoma (44.4–51%) (3), also known as reverse angle-closure glaucoma (4). The etiology of MSP is postulated to be the maldevelopment of the mesoderm (5). The condition may occur in an isolated form or as an ocular manifestation of systematic disorders, including Marfan syndrome (6), Weill-Marchesani syndrome (7), Alport's syndrome (8), cri-du-chat syndrome (9), homocystinuria (10), and chondrodysplasia punctata (11).

The unique morphology and potential complications of MSP challenge the management of the condition. Various surgical approaches have been attempted in sporadic MSP cases, including angle-supported IOL (12), iris-claw IOL (13), retropupillary iris-claw IOL (14), and sutured (15) or sutureless (16, 17) sclera-fixated IOL. However, most of the procedures adapt lensectomy or capsulotomy to remove the capsular bag, and anterior vitrectomy is often requisite. The risk of retinal detachment is expected to increase, especially in those with connective tissue disorders (18). The tilt, decentration, and dislocation of the IOLs are also of great concern (19).

Although the capsular bag of the eyes of MSP patients is relatively small, the preservation of the posterior capsule and residual zonules is still valuable, as they maintain the continuity of the physical barrier between the anterior and posterior segments. Here, we report a novel and feasible surgical procedure, supra-capsular and scleral-fixated intraocular lens implantation (SCSF-IOL), that can overcome some of the challenges in MSP surgery. This study aimed to ascertain the surgical outcomes in a series of consecutive patients with MSP who underwent the SCSF-IOL procedure and compare them with those that received transscleral-fixated modified capsular tension ring and in-the-bag intraocular lens implantation (MCTR-IOL). The visual outcomes and postoperative complications were evaluated to compare the efficacy and safety of these two procedures.

## Methods

### Patient Eligibility and Ethics Statement

Patients with MSP were recruited, all of whom received lens surgery at the Eye and ENT Hospital of Fudan University, Shanghai, China, from Jan 2019 onwards. The MCTR-IOL procedures were performed before June 2020 when the registration certificate of MCTR expired in mainland China. From then on, all MSP eyes underwent the SCSF-IOL procedures. MSP was diagnosed in accordance with a previous study (3). Briefly, MSP was diagnosed if the entire lens equator was observed under complete pupil dilation and, for eyes with limited pupil diameter, anterior segment optical coherence tomography (AS-OCT) was supplemented. The surgical indications were as follows: (1) a best corrected distance visual acuity (BCVA) (LogMAR) worse than 0.5; (2) uncorrectable lenticular astigmatism; (3) pupillary block due to lens dislocation; and (4) a high risk of amblyopia progression. Patients with the following features were not enrolled: (1) lens dislocated into the anterior chamber or posterior pole; (2) a history of eye trauma or intraocular surgery; and (3) the coexistence of retinal detachment, retinal pigmentosa, end-stage glaucoma, or cornea endothelium decompensation. The surgical eyes were registered for patients who undergone unilateral surgery. One of the eyes was randomly selected if the patient was operated bilaterally. All procedures performed on human participants followed the 1964 Declaration of Helsinki and its later amendments after receiving proper approval from the Human Research Ethics Committee of the Eye & ENT Hospital of Fudan University (no. 2020126-1). Informed consent was obtained from all candidates and the guardians of those under 18.

### Ophthalmic Examinations

All enrolled patients underwent slit-lamp examination under complete pupillary dilation by the same experienced ophthalmologist. Their BCVA was measured by an experienced optometrist. The ocular biometry was obtained using partial coherence interferometry (IOLMaster 500 & 700, Carl Zeiss Meditec AG, Jena, Germany). The intraocular pressure (IOP) was measured with a non-contact tonometer (CT-80, Topcon Medical Systems, Oakland, US), and the retro illumination images and ocular aberrations were recorded with a wave front aberrometer (OPD-Scan III, Nidek Co, Ltd., Gamagori, Japan). The tilt of the IOL was obtained indirectly from the wave front aberrations, including tilt, coma, and trefoil, as was previously described (20). The anterior segment was visualized by swept-source AS-OCT (CASIA2; Tomey Corp, Nagoya, Japan).

### Surgical Management

**Figure 1:**
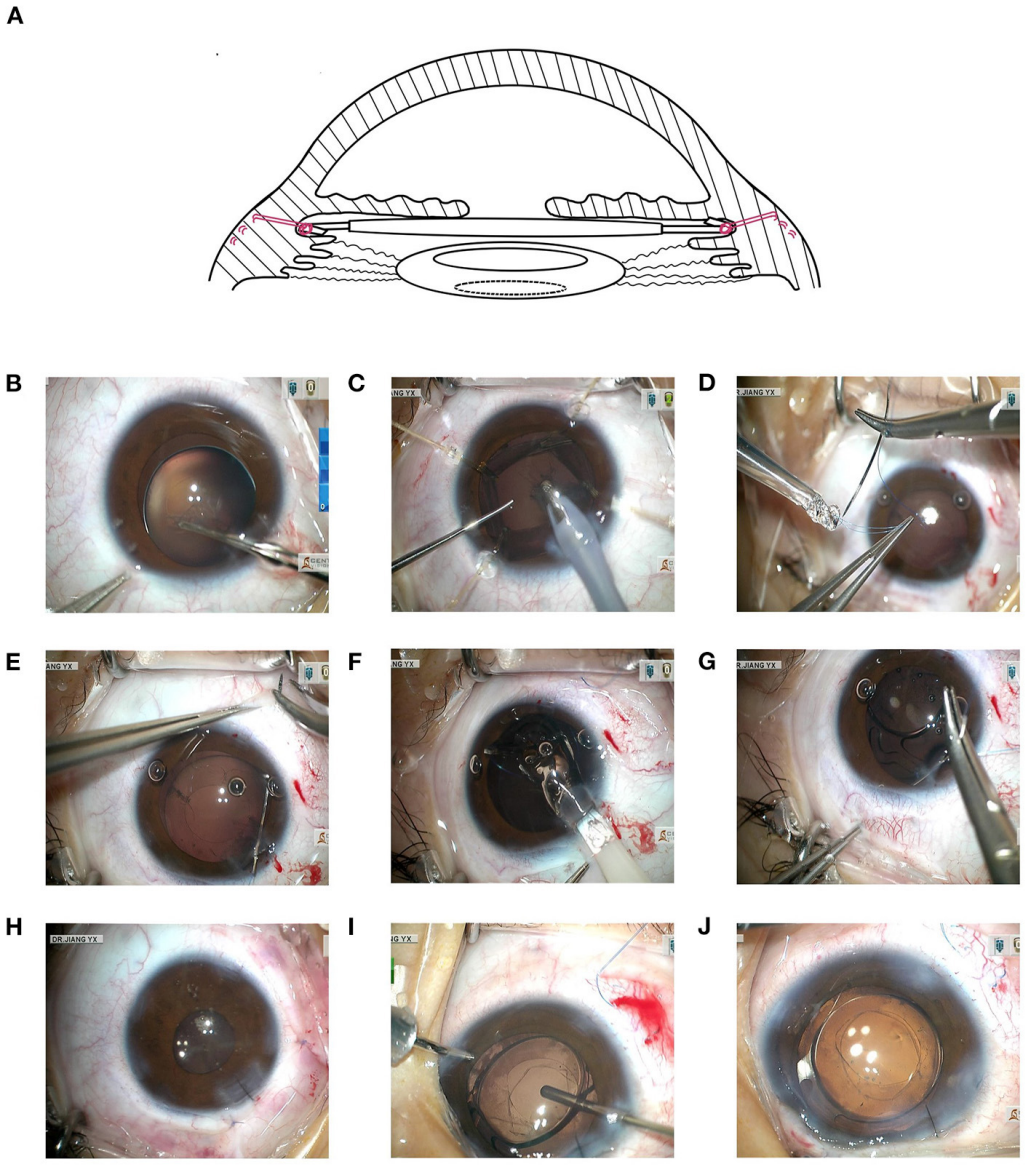
Detailed processes of SCSF-IOL in MSP. **(A)** A demonstration of the principles of the SCSF-IOL procedure. The intraocular lens was sutured with 9–0 polypropylene (in red) through the sulcus and placed above the preserved capsule. Prophylactic posterior capsulotomy is shown within the dashed circle. **(B)** Continuous circular capsulorhexis was carefully performed. **(C)** The lens was removed using irrigation/aspiration (I/A) mode at reduced vacuum, slow aspiration flow rate, and low bottle height, with the aid of four capsular hooks. **(D)** Double-strand 9–0 polypropylene was used to suture one loop of the pre-loaded IOL. **(E)** A puncture point was made using the ab interno approach at 1.5–2.0 mm posterior to the corneal limbus. **(F)** The pre-loaded IOL with the pre-sutured loop was injected into the anterior chamber through a 2.6-mm clear corneal tunnel incision. **(G)** The other loop was sutured opposite to the previous one. **(H)** The main incision and conjunctival flap were closed. **(I)** For young patients who were expected to be uncooperative during laser capsulotomy, the posterior capsule of the visual axis was excised and limited anterior vitrectomy was performed via the limbus with the cutter in cut I/A mode. **(J)** At the center of the IOL, the anterior and posterior capsulorhexis openings were checked at the end of the surgery. This is shown in the same eye as in **(I)**. SCSF-IOL, supra-capsular and scleral-fixated intraocular lens implantation; I/A, irrigation/aspiration; MSP, microspherophakia.

All procedures were performed by the same experienced surgeon (YX.J.) and in the same setting. The step-by-step procedure was shown in Figure 1. The phacoemulsification procedure and MCTR implantation were performed as described in detail in our previous study (21), but four capsular hooks (CapsuleCare, Med Devices Lifesciences, Vaishali, India) were applied to stabilize the bag in cases of MSP. In the SCSF-IOL group, a single-piece IOL (Superflex Aspheric 920H or C-flex Aspheric 970C, Rayner Surgical Group Ltd., West Sussex, UK) was injected into the anterior chamber through a 2.6-mm clear corneal tunnel incision. The loop of the pre-loaded IOL was sutured to the sclera by double-strand 9–0 polypropylene (MANI Inc., Tokyo, Japan) through the sulcus, and the capsular bag was left intact. Z-suturing was applied to fixate the suture in the sclera with no need to generate a scleral flap (22). An 8–0 vicryl polyglactin suture (Ethicon, NJ, USA) was applied to close the conjunctival flap. In the SCSF-IOL group, prophylactic Nd:YAG laser posterior capsulotomy was performed 1 week to 1-month postoperatively. Limited posterior capsulotomy and anterior vitrectomy (23G, Alcon Laboratories Inc., Geneva, Switzerland) were performed intraoperatively using a limbal approach in children who were expected not to cooperate with laser treatment. For patients in the MCTR-IOL group, laser capsulotomy was performed only when posterior capsule opacification or anterior capsule contraction was visually significant.

### Statistical Analysis

Data normality was confirmed using the Shapiro–Wilk test. Student's *t*-test and Mann–Whitney *U* test were applied as appropriate for comparisons between the two independent groups. Descriptive statistics included the mean ± standard deviation and median (interquartile, IQ) where appropriate. The paired Student's *t*-test or paired Wilcoxon test was used to compare preoperative with postoperative measurements within the same group. The results of the two-sided tests were considered significant at *P* < 0.05. Statistical analyses were performed using SPSS version 25w.0 (IBM Corp., Armonk, NY, USA).

## Results

### Preoperative Characteristics

**Figure 2:**
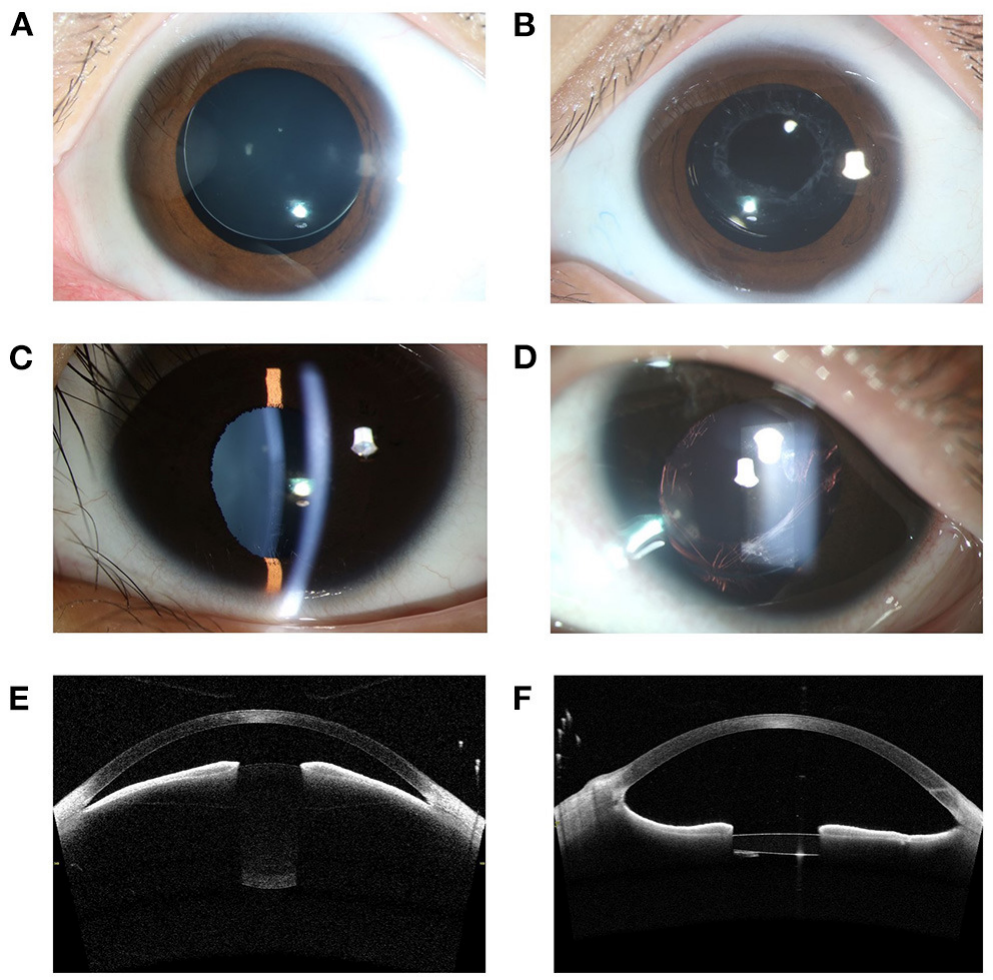
Representative photographic images and AS-OCT images of MSP eyes. **(A)** A slit-lamp photograph of a MSP eye with Marfan syndrome before surgery revealed a small lens with superior dislocation. **(B)** The preserved capsule on 1-year follow-up after Nd:YAG laser treatment. This is the same eye as in **(A)**. **(C)** One eye of MSP was complicated with ectopia pupillae. **(D)** The visual axis was clear on 1-year follow-up after the SCSF-IOL procedure and Nd:YAG laser capsulotomy. This is the same eye as in **(C)**. **(E)** AS-OCT showed the spherically shaped lens and forward migration of the iris-lens diaphragm in one MSP eye with Marfan syndrome. **(F)** The narrowing of the anterior chamber angle was significantly relieved 3-month postoperatively. This is the same eye as in **(E)**. AS-OCT, anterior segment optical coherence tomography; SCSF-IOL, supra-capsular and scleral-fixated intraocular lens implantation; MSP, microspherophakia.

A total of 37 patients (37 eyes) with MSP were recruited. Twenty eyes underwent the SCSF-IOL procedure and 17 eyes received MCTR-IOL. The representative clinical features are shown in Figure 2, and the characteristics of the patients in the two groups are shown in Table 1. The demographic parameters, including sex, clinical diagnosis, age at surgery, preoperative BCVA, and prevalence of cataract and glaucoma, were not significantly different between the two groups.

**Table 1:** Preoperative characteristics of patients with MSP.

**Characteristics**	**Group** ^a^		***P*-value**
	**SCSF-IOL**	**MCTR-IOL**	
No. patients	20	17	
Male/female	12/8	10/7	1.000
**Clinical diagnosis**
Isolated	8	9	0.517
Syndromic (MFS/HCY)	11/1	7/1	
No. eyes	20	17	
Right/left	10/10	10/7	0.743
Age at the surgery (years)	12.00 (5.00, 21.00)	6.50 (5.50, 27.50)	0.562
BCVA (LogMAR)	0.70 (0.40, 0.80)	0.70 (0.40, 1.00)	0.405
IOP (mmHg)	14.57 ± 3.22	15.20 ± 5.28	0.669
Cataract (%)	15.0%	11.8%	1.000
Glaucoma (%)	10.0%	23.5%	0.383
Follow up (month)	4.50 (2.25, 6.50)	4.00 (2.00, 7.00)	0.988

*BCVA, best-corrected visual acuity; HCY, homocystinuria; LogMAR, logarithm of the minimal angle of resolution; IOP, intraocular pressure; MCTR-IOL, transscleral-fixated modified capsular tension ring and in-the-bag intraocular lens implantation. MFS, Marfan syndrome; MSP, microspherophakia. SCSF-IOL, supra-capsular and scleral-fixated intraocular lens implantation*.

a *Normally distributed data are shown in the mean ± standard deviation, while skewed data are shown in median (interquartile)*.

### Postoperative Surgical Outcomes

**Figure 3:**
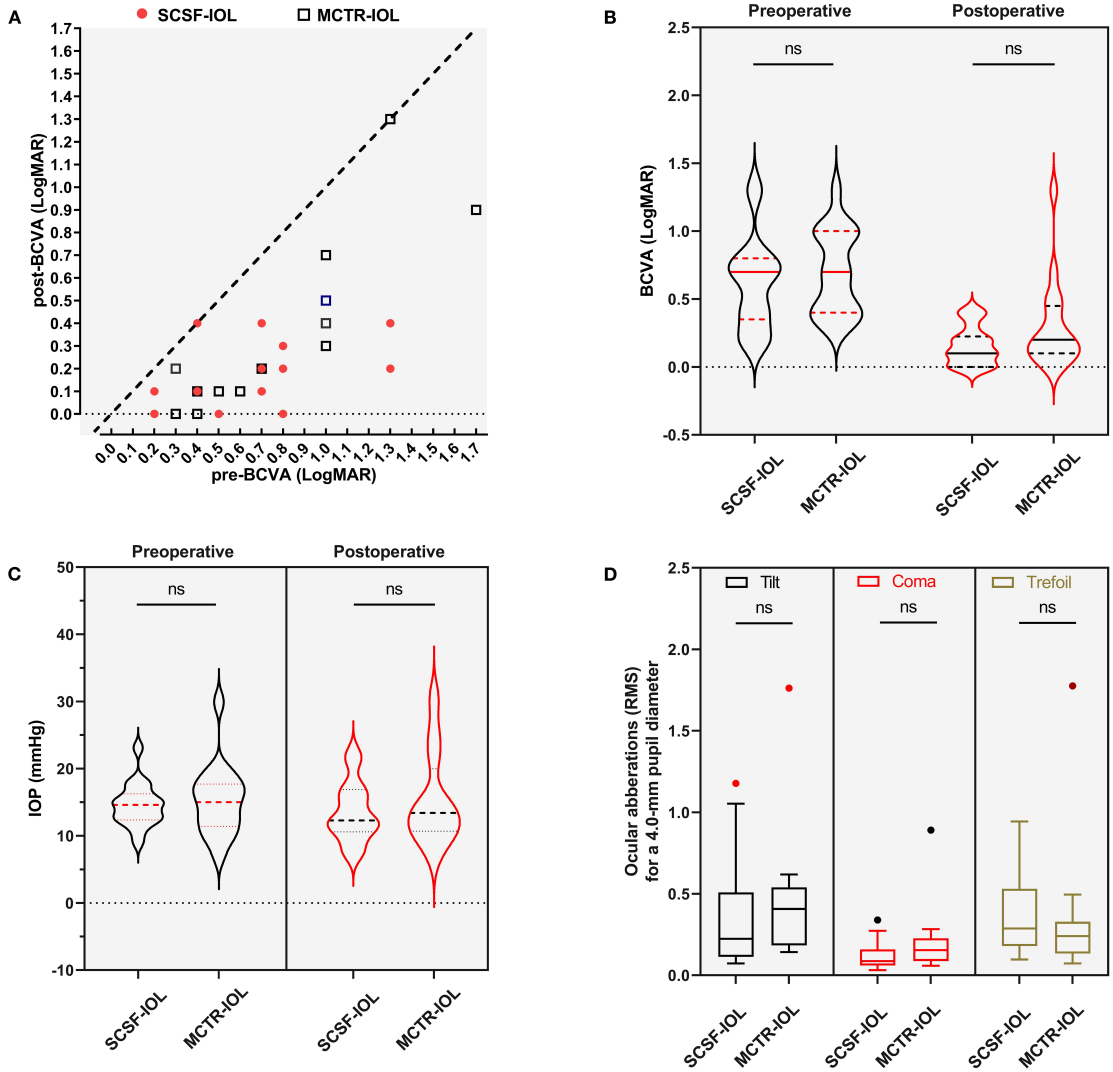
Comparison of surgical outcomes of SCSF-IOL and MCTR-IOL in eyes with MSP. **(A)** Scatterplot of preoperative and postoperative BCVA on final follow-up in the SCSF-IOL (red dots) and MCTR-IOL (black square) groups. **(B)** Nested violin graph of preoperative and postoperative BCVA on final follow-up in the SCSF-IOL and MCTR-IOL groups. The medians are shown in solid lines, and the interquartiles are presented as dashed lines. **(C)** Nested violin graph of preoperative and postoperative IOP on final follow-up in the SCSF-IOL and MCTR-IOL groups. The medians are shown in solid lines, and the interquartiles are presented as dashed lines. **(D)** Comparison of postoperative ocular aberration (tilt, coma, and trefoil) between SCSF-IOL and MCTR-IOL groups. BCVA, best-corrected visual acuity; IOP, intraocular pressure; SCSF-IOL, supra-capsular and scleral-fixated intraocular lens implantation; LogMAR, logarithm of the minimal angle of resolution; MCTR-IOL, transscleral-fixated modified capsular tension ring and in-the-bag intraocular lens implantation. RMS, root mean square.

The patients were followed up for a similar duration in the two groups (Table 1). The patient's BCVA was evaluated at the last follow-up. Most of the eyes showed improved BCVA, and the difference was significant in both the SCSF-IOL group (paired *t* test, *P* < 0.001) and the MCTR-IOL group (paired *t* test, *P* < 0.001) (Figure 3A). The BCVA (LogMAR) at last follow-up was 0.13 (IQ: 0.02, 0.28) in the SCSF-IOL group and 0.15 (IQ: 0.10, 0.46) in the MCTR-IOL group, and the difference between the two groups was insignificant (Mann–Whitney test, *P* = 0.326) (Figure 3B). Both of the procedures didn't lower the IOP (SCSF group, paired Wilcoxon test, *P* = 0.196; MCTR group, paired Student's *t*-test, *P* = 0.824) and the postoperative IOP is similar between the two groups (Mann–Whitney test, *P* = 0.755) (Figure 3C). No secondary glaucoma was observed after the surgery. The Ocular aberrations were evaluated to indirectly compare the severity of IOL tilt between the two groups. The tilt (Mann–Whitney test, *P* = 0.216), coma (Mann–Whitney test, *P* = 0.151), and trefoil (Mann–Whitney test, *P* = 0.264) were not significantly different between the two groups (Figure 3D and Supplementary Table 1).

### Postoperative Capsule Changes and Complications

**Figure 4:**
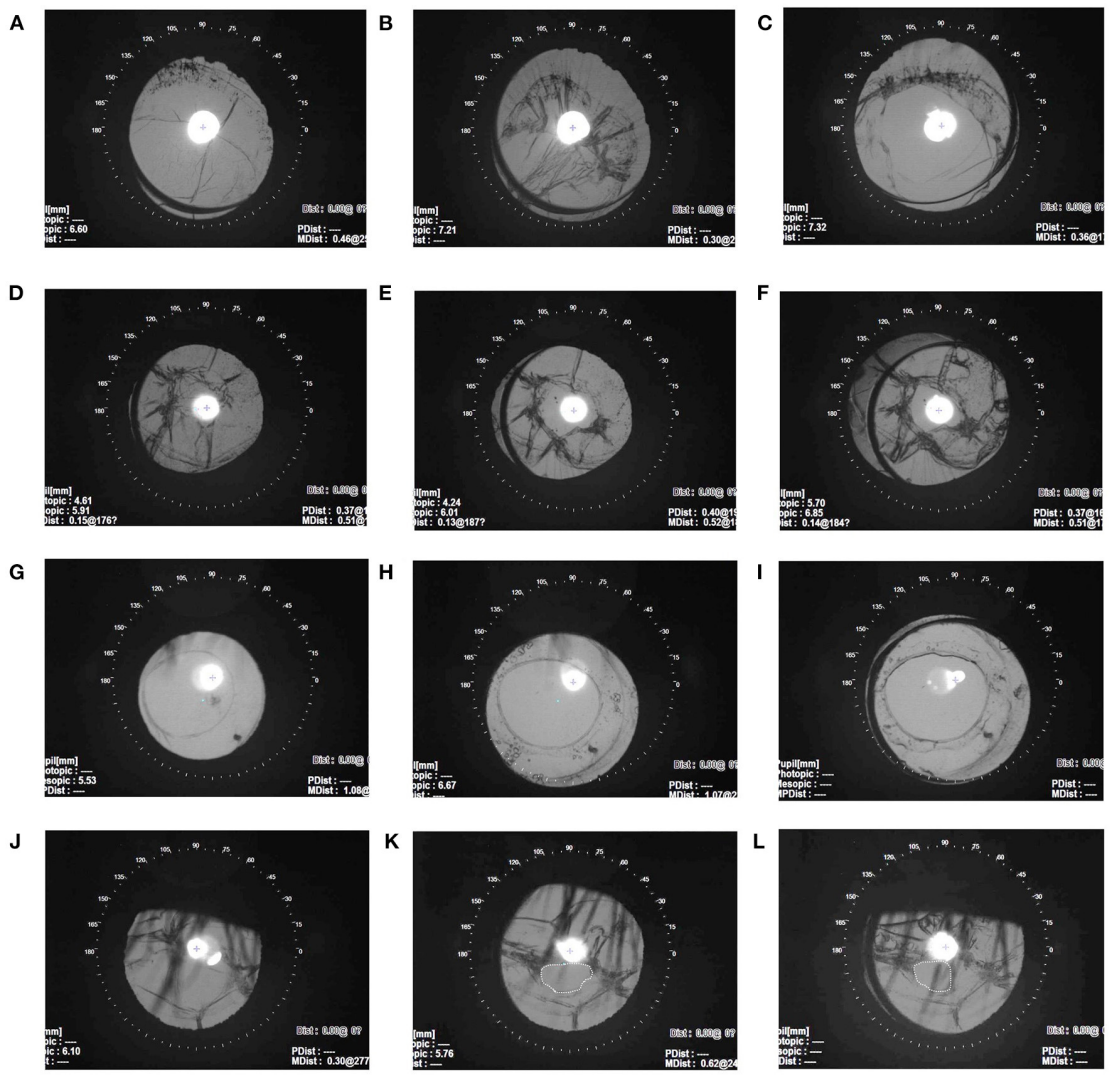
Postoperative capsule change in the SCSF-IOL group. **(A–C)** The retro illumination images show residual capsule postoperative changes in the same MSP eye. The capsule was clear and flat 1 week after surgery **(A)** and contracted 1-month postoperatively **(B)**. After laser treatment for 1 month, the contraction was ameliorated, and the visual axis was clear **(C)**. **(D–F)** Capsule changes in the same MSP eye 1 day before Nd:YAG laser capsulotomy **(D)** and 1 month after laser treatment **(E)**. The opening remained centered, and the BCVA achieved 0.0 LogMAR at 1-year follow-up **(F)**. **(G–I)** A 5-year-old boy with MSP underwent regional posterior capsulotomy and limited anterior vitrectomy during the surgery. The capsule remained stable at the 1-week **(G)**, 6-month **(H)**, and 9-month **(I)** follow-up visits. **(J–L)** A 12-year-old girl with MSP had an unexpected decentered posterior capsulorhexis opening (dashed circle). The retro illumination images show the capsule before laser treatment **(J)**. The posterior capsule opening was decentered 5 months after laser capsulotomy **(K)** and 1 year after surgery **(L)**. The BCVA was 0.4 LogMAR at 1-year follow-up. BCVA, best-corrected visual acuity; SCSF-IOL, supra-capsular and scleral-fixated intraocular lens implantation; LogMAR, logarithm of the minimal angle of resolution; MSP, microspherophakia.

In the SCSF-IOL group, the preserved capsule was clear and flat 1 week postoperatively (Figure 4A) and began shrinking 1 month after surgery (Figure 4B), and prophylactic laser capsulotomy was applied to clear the visual axis (Figure 4C). The position of the posterior capsulorhexis opening can be seen to be steady in some patients on a 1-year follow-up (Figures 4D–F). Two eyes (10.00%) were laser-treated twice to achieve a satisfying posterior capsulorhexis opening, all of whom were under 8 years old. For two young patients who had undergone regional posterior capsulotomy and limited anterior vitrectomy during the operation, the capsule opening remained centered with minimal contraction, and no further laser treatment was required (Figures 4G–I). One eye (5.00%) had an unexpectedly decentered capsule opening after laser treatment (Figures 4J–L). In the MCTR-IOL group, significant visual posterior capsular opacification (PCO) developed postoperatively in six eyes (35.29%), which were treated by Nd:YAG laser capsulotomy. Excepting transient visual complaints of floating material from several patients, no other complications were recorded after laser treatment in both groups. Two eyes (10.00%) in the SCSF-IOL group and 4 eyes (23.53%) in the MCTR group were diagnosed with glaucoma before surgery. All cases demanded anti-glaucoma eye drops postoperatively but in a less intensive manner. No secondary glaucoma was observed in either group. No incidences of suture exposure, IOL dislocation, cystoid macular edema, or retinal detachment were recorded in either group.

## Discussion

The high risk of lenticular ametropia and complications secondary to lens dislocation threaten the long-term visual prognosis of patients with MSP. Early surgical intervention is recommended, but lens extraction plus IOL implantation is challenged by the combined effects of insufficient zonules and undersized capsules. Currently, we are lacking a gold standard treatment for MSP patients. In this study, we demonstrated a novel and minimally invasive procedure, SCSF-IOL, which involves suturing the IOL through the sulcus without complete capsulotomy or vitrectomy. The SCSF-IOL procedure resulted in significant visual improvements, fine IOL stability, and a tolerant range of complications. Though the results show insignificant difference when compared to that of the MCTR-IOL group, we proposed that the SCSF-IOL procedure is simple and practicable for the surgeons familiar with anterior approaches.

Various approaches are available for the surgical removal of a dislocated lens. In the 1970s, intracapsular or extracapsular removal had a high incidence of vitreous loss and retinal detachment in eyes with ectopia lentis (23, 24). With the development of medical instruments, phacoemulsification has gained popularity as a technique for removing a dislocated lens, but it was considered difficult to use in the eyes of MSP, probably because of the severe loss of capsular support (25). Thus, surgeons have tended to not save the capsular bag and perform capsulotomy and vitrectomy. However, the preservation of the capsular bag and residual zonules is worthy, as this leaves the posterior segments intact, which minimizes the risk of vitreoretinal complications, such as retinal detachment, vitreous prolapse, suprachoroidal, and vitreous hemorrhage. The incidence of retinal detachment of scleral fixated IOL after capsulotomy and anterior vitrectomy was relatively high (4.1–17.2%) (18, 26–29), but it was less common in capsule-reserved procedures such as capsular tension ring (CTR), capsular tension segment (CTS), and MCTR (0.00–2.40%) (30–32). Meanwhile, the preserved capsular bag and intact anterior vitreous body were likely to provide additional support to secure the position of IOL and thus reduce the possibility of the IOL falling into the vitreous body (33, 34). Thus, it is reasonable that no retinal detachment nor IOL dislocation was observed in this study and the tilt of IOLs was comparable in both groups. One published study reported the application of a similar capsule-reserved approach in MSP (35). The surgeon persevered the anterior capsule leaves and incarcerated them with the optic region of the sulcus-implanted IOL without suturing. However, we postulated that suturing of the IOL is essential in eyes with MSP, considering the limited support provided by the residual capsule, especially when the bag has not undergone fibrosis or zonule weakness becomes progressive.

In addition to lens removal, the other issue is how to fix the IOL properly in the setting of insufficient capsular support. The success of in-the-bag IOL implantation stabilized by a CTR has been reported for cases of MSP (36). With the aid of capsular hooks, the CTR can be delivered uneventfully in the small and unset capsular bag and is well-tolerated. However, a sutureless CTR might not be stable in MSP, in which the zonular weakness covers 360-degrees and is very likely to be progressive. Thus, in several cases, CTS together with CTR was implemented and sutured to the scleral (37, 38), which exerted almost the same effect as MCTR. Although MCTR has been widely used in eyes of ectopia lentis patients, the application of MCTR in MSP has been reported in a limited number of studies. One case study reported using single-eyelet MCTR together with CTS to achieve two-point scleral suturing in one eye of MSP (39). The authors did not employ two-eyelet MCTR, as they thought this technique was difficult and less repeatable (39). We agreed with the perspective that it is technically demanding to implant the standard size MCTR in the already small and lax bag. The tearing of capsulorhexis may happen during the MCTR or IOL implantation and the surgeon has to perform the capsulotomy and deal with the prolapsed vitreous. Our study showed similar visual outcomes in MSP patients in both the SCSF-IOL and MCTR-IOL groups. However, the SCSF-IOL procedure is relatively simple. Another relatively large case series also reported the use of MCTR in three eyes of MSP with good postoperative stability; however, the author still favored the anterior chamber IOL due to its easy availability and affordability (12). In our opinion, the most common complication is bullous keratopathy when it comes to the anterior chamber IOLs, and some less common but potentially devastating complications should be considered, such as macular edema, secondary glaucoma, and IOL dislocation (40). A larger corneal incision was also required for the anterior chamber IOL implantation compared to that of this study (41). Hence, we recommended the use of posterior chamber IOL and the pre-loaded system. Scleral fixated IOLs were associated with conjunctival erosion (42). However, using the knotless Z-suture technique, the complications related to a scleral flap and exposed knots are becoming less common. Furthermore, the knotless and double-strand 9-0 polypropylene may also contribute to the stability of the fixation.

Capsule opacification and contraction are almost inevitable with the SCSF-IOL procedure due to the preservation of the capsular bag without contact with the optic region of the IOL. Therefore, Nd: YAG laser capsulotomy is routinely prescribed to prevent the contraction or clouding of the capsule in the visual axis 1-month after the SCSF-IOL procedure, and as early as 1-week for young patients. Though some children underwent repeated laser capsulotomy, satisfying laser capsulorhexis was achieved in most eyes. The Nd: YAG laser capsulotomy was proved to be both effective and safe in young patients (43), however, considering the risk of poor cooperation of some young patients, regional capsulotomy and limited vitrectomy were applied in the primary surgery. One eye, unfortunately, had a small and decentered opening, probably due to the asymmetric weakness of the zonules or contraction of the fibrotic capsule. Therefore, we propose that the eyes of ectopia lentis without MSP or MSP complicated by severe lateral dislocation may not benefit from the SCSF-IOL procedure. The asymmetric force from the residual zonules is likely to make the position of the capsular bag unpredictable, and visual acuity could be compromised once the equatorial capsule blocks the visual axis, which can be refractory to the laser capsulotomy.

Glaucoma is another concern in eyes of MSP, but the incidence of glaucoma before the surgery was lower than that in the existing literature (4, 44). This is probably because the enrolled patients were relatively young and their peripheral anterior synechiae had not yet developed. Although lens extraction plus IOL implantation could ameliorate the crowding of the anterior chamber, some patients still need adjunctive medication to control the IOP. This finding is consistent with previous studies that found anti-glaucoma medicine is necessary for some patients with MSP despite lens surgery (36, 44). In addition, we did not exclude the possibility that angle dysplasia coexisted with MSP in some individuals (4).

The drawbacks of the study included its retrospective design, limited duration of follow-up, and relatively small cohort size. However, considering the rarity of this disease, this is one of the largest studies that has focused on the surgical management of MSP. The long-term curative effects and late-onset complications of the two capsule-reserved approaches remain to be recorded during further follow-up. Despite the above limitations, we feel that our investigation may contribute to the development of appropriate surgical options in the setting of MSP.

In conclusion, the current study, involving a relatively large number of consecutive patients with MSP, reported the efficacy of a novel technique, SCSF-IOL, which removes lens material by phacoemulsification and preserves the residual capsule in a relatively simple way. This procedure resulted in a good prognosis and limited complications, comparable to those achieved by MCTR-IOL. We consider the SCSF-IOL procedure to be a feasible option for the treatment of MSP, especially for anterior surgeons.

## Data Availability Statement

The original contributions presented in the study are included in the article/Supplementary Material, further inquiries can be directed to the corresponding authors.

## Ethics Statement

All procedures performed on human participants followed the 1964 Declaration of Helsinki and its later amendments after receiving proper approval from the Human Research Ethics Committee of the Eye & ENT Hospital of Fudan University (no. 2020126-1). Informed consent was obtained from all candidates and the guardians of those under 18. Written informed consent to participate in this study was provided by the participants' legal guardian/next of kin. Written informed consent was obtained from the individual(s), and minor(s)' legal guardian/next of kin, for the publication of any potentially identifiable images or data included in this article.

## Author Contributions

Z-XC and Z-NZ conceived and designed the study. YS, W-NJ, J-LZ, J-HC, T-HC, and L-NL collected the clinical samples. Y-XJ performed clinical examinations of patients and clinical interpretation. Z-XC and Z-NZ drafted and revised the manuscript. Y-XJ supervised the whole project and provided critical reviews. All authors read and approved the manuscript.

## Funding

This study was supported by the Shanghai Science and Technology Commission (Grant no. 20Y119110), the Shanghai Science and Technology Commission (Scientific Innovation Action Plan, Grant no. 18411965200), the National Key R&D Program (Grant no. 2018YFC0116004), and the National Natural Science Foundation of China (Grant no. 82070943).

## Conflict of Interest

The authors declare that the research was conducted in the absence of any commercial or financial relationships that could be construed as a potential conflict of interest.

## Publisher's Note

All claims expressed in this article are solely those of the authors and do not necessarily represent those of their affiliated organizations, or those of the publisher, the editors and the reviewers. Any product that may be evaluated in this article, or claim that may be made by its manufacturer, is not guaranteed or endorsed by the publisher.
