# Supplementary material for: Phacoemulsification Combined With Supra-Capsular and Scleral-Fixated Intraocular Lens Implantation in Microspherophakia: A Retrospective Comparative Study
Source: Front Med (Lausanne). 2022 Apr 14;9:869539. doi: 10.3389/fmed.2022.869539 (PMC9047048; doi:10.3389/fmed.2022.869539)
Supplement: Supplementary file 1 [file Table_1.docx]

**Supplementary Table 1. Comparison of surgical outcomes of SCSF-IOL and MCTR-IOL in eyes with MSP.**

|  | Preoperative^a^ | | *P* value | Postoperative^a^ | | *P* value |
| --- | --- | --- | --- | --- | --- | --- |
|  | SCSF-IOL | MCTR-IOL |  | SCSF-IOL | MCTR-IOL |  |
| BCVA  (LogMAR) | 0.70 (0.40, 0.80) | 0.70 (0.40, 1.00) | 0.405 | 0.13 (0.02, 0.28) | 0.15 (0.10, 0.46) | 0.326 |
| IOP  (mmHg) | 14.57 ± 3.22 | 15.20 ± 5.28 | 0.669 | 12.3 (10.60, 16.90) | 13.4 (10.7, 19.98) | 0.755 |
| Tilt  (RMS) | - | - | - | 0.22 (0.20, 0.52) | 0.41 (0.18, 0.54) | 0.216 |
| Coma  (RMS) | - | - | - | 0.09 (0.06, 10.16) | 0.15 (0.09, 0.23) | 0.151 |
| Trefoil  (RMS) | - | - | - | 0.29 (0.18, 0.53) | 0.24 (0.13, 0.33) | 0.264 |

BCVA, best-corrected visual acuity; IOP, intraocular pressure; SCSF-IOL, supra-capsular and scleral-fixated intraocular lens implantation; LogMAR, logarithm of the minimal angle of resolution; MCTR-IOL, transscleral-fixated modified capsular tension ring and in-the-bag intraocular lens implantation. RMS, root mean square.

a. Normally distributed data are shown in the mean ± standard deviation, while skewed data are shown in median (interquartile).
